# The nephrotoxin ochratoxin a impairs resilience of energy homeostasis of human proximal tubule cells

**DOI:** 10.1007/s12550-023-00500-7

**Published:** 2023-07-19

**Authors:** Gerald Schwerdt, Michael Kopf, Michael Gekle

**Affiliations:** Julius-Bernstein-Institut Für Physiologie, Martin-Luther-Universität Halle-Wittenberg, Magdeburger Str. 6, 06112 Halle, Germany

**Keywords:** Ochratoxin A, Mitochondria, Energy metabolism, Glucose

## Abstract

Despite a long history of research, the mode of action of the mycotoxin ochratoxin A (OTA) is still not clear. Based on our observation that OTA-exposed cells consume more glucose and produce more lactate than control cells, with this study, we want to suggest another possible mode of action of OTA, involving cellular metabolism and mitochondria. We exposed human proximal tubule cells (HK2 cells) to OTA and studied its influence on mitochondrial performance as well as on the expression of energy homeostasis-involved routing proteins (AMPK and TXNIP) and on glucose transporting and metabolizing proteins. OTA reduced the capacity of mitochondria to increase their oxygen consumption rate forcing the cells to switch to the ineffective anaerobic glycolysis which demands higher glucose availability. The higher glucose demand is met by augmented cellular glycogen degradation and increased glucose uptake capabilities by increasing glucose transporter expression. We conclude that OTA exposure leads to impaired mitochondria, which forces the cells to alter their metabolism in order to ensure energy supply. We suggest to consider a possible effect of OTA on metabolism and mitochondria and to have a closer look on OTA-induced changes in the metabolome as possible additional players in OTA toxicity.

**Supplementary Information:**

The online version contains supplementary material available at 10.1007/s12550-023-00500-7.

## Introduction

The enigma how the mycotoxin ochratoxin A (OTA) leads to cellular dysfunction or damage is still not solved (Malir et al. 2016). OTA is found in a wide assortment of human food (Arce-Lopez B et al. 2020), and therefore, its uptake is almost unavoidable (Duarte et al. 2010). One organ affected by OTA is the kidney and therein especially the proximal tubuli where at least two major cell types are endangered by OTA: the epithelial cells and the fibroblasts although the latter supposedly not as serious (Schwerdt et al. 2007). These two cell types obviously interact with each other by some kind of cross-talk thereby influencing the severity of OTA toxicity (Schulz et al. 2019; Schwerdt et al. 2021). Many mechanisms have been proposed how OTA may act on cells: one of the first ideas considered an interaction with phenylalanine-handling proteins because of the phenylalanine moiety of OTA. This should lead for example to impaired ribosomal protein expression (Creppy et al. 1984). A study using synthesized OTA derivatives confirmed that the phenylalanine moiety is indeed necessary for the interaction of OTA with its—still unknown—target molecules (Rottkord et al. 2017). Furthermore, DNA adducts have been supposed as another mechanism to explain the renal carcinogenesis of OTA (Pfohl-Leszkowicz and Manderville 2012). Often reactive oxygen species are discussed to play a role in OTA toxicity besides a bundle of assumed additional modes of action (Khoi et al. 2021a, b) including signaling pathways (Benesic et al. 2000; Gekle et al. 2000) and influence on post-transcriptional regulation by micro- and long non-coding RNAs (Hennemeier et al. 2014; Polovic et al. 2018).

One effect of OTA exposure is apoptosis (Schwerdt et al. 1999), which together with altered collagen homeostasis leads to tubule damage (Schwerdt et al. 2007). A mitochondrial involvement in the onset of apoptosis is well documented (Jeong and Seol 2008; Kroemer and Reed 2000) as e.g. mitochondrial cytochrome c release which leads to enhanced caspase-3 activity. The main role of mitochondria in energy homeostasis is to ensure a sufficient level of cellular ATP. Restricted mitochondrial function therefore, is a severe threat to the cells forcing them to use other possibilities for ATP production. An exception of this finding can be seen in tumor cells, which often use anaerobic glycolysis as ATP source with formation of lactic acid which in turn leads to acidification of the tumor tissue. Intra-and extracellular acidification was shown to prevent OTA-induced cell death in a proximal tubule-derived cell line (Schwerdt et al. 2003). The cellular energy demand is fine-tuned by several mechanisms. AMP-activated protein kinase (AMPK) is called a sensor of cellular energy level (Lin and Hardie 2018). In case of low energy conditions, AMPK phosphorylates key factors in order to restore energy balance. It inhibits anabolism and stimulates catabolism. It counteracts the effects of mTOR (mechanistic target of rapamycin) leading to decreased cell growth and protein synthesis and thereby to less energy demand. By phosphorylation of targets involved in the trafficking of glucose to increase glucose uptake into cells, AMPK also stimulates glucose utilization. The AMPK-induced degradation of thioredoxin-interacting protein (TXNIP), “a master regulator for glucose homeostasis” (Yoshihara 2020), indirectly increases the amount of the glucose transporter GLUT1. To produce energy, AMPK also stimulates the breakdown of energy-storing macromolecules. In a previous study, we could already show that the mRNA expression of the glycogen-degrading enzyme is up-regulated by OTA as well as the expression of mRNA coding for the glucose transporter GLUT1 whereas the expression of mRNA coding for glycogen synthase was reduced (Schwerdt et al. 2021). This indicates that due to OTA exposure the cells not only use their own glucose store but also additionally enable an increased glucose uptake. AMPK also promotes mitochondrial biogenesis by controlling the mitochondrial number by several mechanisms (Herzig and Shaw 2018). Based on the observation that in human proximal tubule-derived cells (HK2 cells), OTA exposure leads to enhanced glucose consumption and lactic acid formation suggesting impaired mitochondrial function; in the present study, we examined the reaction of some mitochondrial parameters to OTA exposure in the human proximal tubule-derived cell line, HK2-cells. Therefore, the oxygen consumption rates after OTA exposure were studied and the expression of genes coding for mitochondrial proteins (TFAM, a key mitochondrial transcription factor and POLRMT, the mitochondrial RNA polymerase (Scarpulla 2008)) and involved in cellular energy homeostasis were determined. Additionally, the amount of mitochondria was determined by comparing the mitochondrial with the nuclear DNA copy number. We conclude that OTA exposure leads to a weakening of mitochondria forcing the cells to alter their metabolism in order to ensure sufficient energy supply. Therefore, impairment of mitochondria may be—besides the already supposed ones—an additional player to consider in OTA toxicity.

## Material and methods

### Cell culture

Human proximal tubule cells (HK2-cells) were purchased from ATCC (Rockville, USA; CRL-2190). They were cultured in DMEM-HamF12 media (PAN Biotech, Aidenbach, Germany) containing 10% fetal calf serum. Media were changed every week. Twenty-four hours prior to and during OTA exposure, cells were held in serum-free media to exclude uncontrollable effects of serum components. We chose 100 nM OTA to be close to the concentrations expectable during kidney failure and to be far below the micromolar ranges often used in previous studies. OTA (100 nM) led to a slight decrease of protein content mostly due to enhanced apoptosis rates (Schwerdt et al. 2021).

### Determination of glucose and lactate content of media

Glucose and lactate in media were determined photometrically. For glucose determination, 5 µl (out of 4 ml media) were added to 100 µl reagent containing 1.5 mM NADP, 1 mM ATP, 1 U/ml hexokinase, and 1 U/ml glucose-6-phosphate dehydrogenase (Sigma). After 15-min incubation at room temperature, the NADPH-dependent absorbance was determined at 340 nm in a photometer. Glucose concentrations in media were calculated using a glucose standard in a range from 0 to 15 mM.

For determination of lactate in media, 10 µl media were added to 200 µl LDH-containing hydrazine-glycine-buffer (400 mM hydrazine-hydrate, 500 mM glycine, 1 U/ml LDH, 3.2 mM NAD, pH 9.0) and incubated for 30 min at 37 °C. Absorbance was determined at 340 nm in a photometer, and lactate content was calculated using a lactate standard in a range from 0 to 10 mM.

### Determination of glycogen content

Intracellular glycogen was determined according to (Bergmeyer and Bernt 1974). Cells were washed three times, collected and pelleted (10 min, 300 × g). One hundred microliters of 3.6% perchloric acid were added to the pellet, mixed and incubated on ice for 10 min or overnight. Thereafter, 50 µl 1 M KHCO_3_ were added, mixed, and centrifuged (10 min, 12,000 × g). To the supernatant, 250 µl enzyme solution [96 U amyloglucosidase (Sigma) per ml acetate buffer (0.46% acetic acid and 120 mM Na-acetate, pH 4.8)] were added and incubated for 2 h at 40 °C to degrade the glycogen to glucose. This glucose was determined as described above using 5 µl of the solution. Intracellular glucose was determined using the same protocol except the enzymatic glycogen degradation step and was subtracted from the glycogen-generated glucose although the intracellular glucose content was almost undetectable. The results obtained for glucose, lactate, and glycogen were related to total protein content determined in an aliquot taken from the collected cells [lysed in MOPS-Triton buffer (20 mM 3-(N-morpholino)propanesulfonic acid, pH 7.4, 0.1% Triton X100)] using bicinchoninic acid as described in (Lane et al. 1986).

### Oxygen consumption rate

The determination of the cellular oxygen consumption rate was done using the Agilent Sea Horse XF Cell Mito Stress Kit and a Seahorse Agilent XF96 Pro Analyzer following their instructions (Agilent Technologies, Waldbronn, Germany). Fifteen thousand cells were seeded in each well of a Seahorse analyzer plate. After incubation with or without OTA, cells were washed and incubated in XF calibrant at 37 °C for 1 h at environmental CO_2_ pressure. During the measurement procedure, 2 µM oligomycin, 1 µM FCCP (carbonylcyanide-p-trifluoromethoxyphenylhydrazone), and 0.5 µM rotenone/antimycin A/6 µM Hoechst33342 dye (Thermo Scientific) were added succesively. Number of cells in a well was determined by Hoechst 33342 dye in a Tecan Infinite fluorometer (Männedorf, Switzerland)) at 350 nm excitation and 465 nm emission.

### Determination of mitochondrial and nuclear DNA copy numbers

Washed cells were collected and lysed with TRIzol reagent (1 ml, Life Technologies, Darmstadt, Germany) and transferred into a reaction tube. After addition of chloroform (200 µl) and centrifugation (10 min, 12,000 × g), the upper phase was discarded and 300 µl ice-cold 96% ethanol were added. After centrifugation (5 min, 2000 × g), pellet was dissolved at 50 °C in 100 µl water. The amount of the mitochondrial and nuclear DNA copy numbers were determined using ddPCR (Bio-Rad Laboratories GmbH, Feldkirchen, Germany) and the mtDNA/ncDNA ratio was calculated. Mitochondrial-encoded cytochrome c oxidase 2 (MT-CO2) served as mitochondrial DNA representative and and ribosomal protein lateral stalk subunit P0 (RPLP0) as nuclear DNA representative.

### Real-time-PCR

Cells were washed and thereafter lysed with TRIzol reagent and transferred into a reaction tube. After addition of chloroform (200 µl) and centrifugation (12,000 × g), the upper phase was collected and mixed with ice-cold isopropanol. After centrifugation (12,000 × g), the supernatant was removed and the pellet washed twice with 75% ethanol and finally solved in water. Reverse transcription was performed using a commercial kit from Invitrogen (Thermo Fisher Scientific, Waltham, MA, USA) according to their instructions. Real-time PCR was performed using a SYBR Green reagent (Invitrogen). Primers were synthesized by Microsynth AG, Balgach, Switzerland. Primer sequences are given in Table 1. Fold change of gene expression was calculated by the 2^ΔΔCt^ method using the expression of EEF2 and RPS17 as references.

**Table 1:** Primer sequences (in 5´–3´) used in PCR

Gene	Forward	Reverse	Product length
EEF2	GGAGTCGGGAGAGCATATCA	GGGTCAGATTTCTTGATGGG	108
SLC2A1 (GLUT1)	ACACTGGAGTCATCAATGCC	ACACTGGAGTCATCAATGCC	148
SLC2A3 (GLUT3)	TTCCACGCTCATGACTGTTT	AGTTCGGCCACAATAAACCA	141
HK2	ATTCGCACTGAGTTTGACCA	TGGCCATCTTCACCAGGATA	127
MT-CO2	TCTTGCACTCATGAGCTGTC	TGAAACTGTGGTTTGCTCCA	144
POLRMT	AGTGCCTCTTTGAGAAGCAG	AGTGCTTTCTCCCATTGGTC	145
PRKAA1	TTGTCACAGGCATATGGTGG	ATAGTTGGGTGAGCCACAAC	151
PRKAA2	AATGTCCTGTTGGATGCACA	CCTGAGATGACTTCAGGTGC	131
PRKAB1	GGGCGGAAAGGAAGTTTACT	GTCCACTGACCATCCACAAA	144
PRKAB2	GTTTGTATCATGGCAGCAGGAT	GAACTTGTATTGGTGCTCTCCC	208
RPLP0	AGTGATGTGCAGCTGATCAA	AAGGAGAAGGGGGAGATGTT	89
RPS17	TCAGCCTTGGATCAGGAGAT	CATCCCAACTGTAGGCTGAG	114
SLC5A2 (SGLT2)	AGGAAAGGGGGAATGAGACT	AAGTCAGAAGCAGGACCAAC	133
TFAM	CCGAGGTGGTTTTCATCTGT	ACGCTGGGCAATTCTTCTAA	147
TXNIP	CTCGTGTCAAAGCCGTTAGGA	TCACCATCTCATTCTCACCTGT	153
UCP1	AGGTCCAAGGTGAATGCCC	TTACCACAGCGGTGATTGTTC	75

### Western blots

Proteins were separated by sodiumdodecylsulfate-polyacrylamide gel electrophoresis (SDS-PAGE) and transferred onto a nitrocellulose membrane. Free binding sites of the membrane were blocked by a 5% solution of non-fat dry milk in TRIS-buffered saline (3 mM TRIS base, 140 mM NaCl, 0.17 mM Tris–HCl, pH 7.4) containing 0.1% Tween20. First antibodies were diluted in TRIS-buffered saline + 5% bovine serum albumin (TRIS-BSA, for dilutions, see Table 2) were added and membranes incubated overnight. After washing, fluorescence-coupled secondary antibodies in TRIS-BSA were added for 90 min. Fluorescence of the second antibodies was recorded using a LI-COR detection system (LI-COR Biosciences, Bad Homberg, Germany).

**Table 2:** Antibodies used in western blot experiments

Antibody against	Source	Dilution
TXNIP	Cell Signaling	1:1000
AMPK	Cell Signaling	1:500
pAMPK	Cell Signaling	1:500
GLUT1	Cell Signaling	1:1000
GAPDH	Cell Signaling	1:1000
Mouse antibody	LI-COR	1:40,000
Rabbit antibody	LI-COR	1:40,000

### Statistics

The significance of difference was determined by the unpaired Student’s *t* test. *p* ≤ 0.05 was considered to be statistically significant and indicated by an * in the figures.

## Results

### Amount of glucose and lactate in media and of intracellular glycogen

**Fig. 1:**
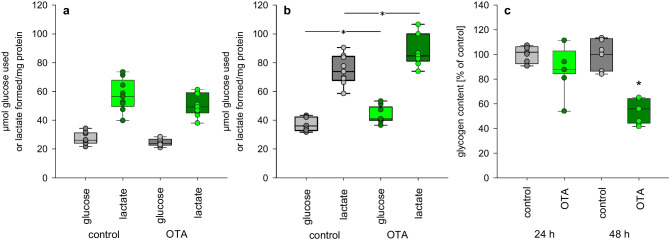
Glucose consumption, lactate production, and glycogen content in HK2 cells after exposure to 100 nM OTA. Lactate and glucose consumption after **a** 24 h and **b** after 48 h; **c** glycogen content after 24 and 48 h. *N* = 3, *n* = 9. *p < 0.05

We determined the glucose and lactate content in media to get an idea on the metabolic condition in which the cells are when exposed to OTA. The media originally contains 5.5 mM glucose and 4 mM glutamate as possible source of lactate. As shown in Fig. 1, after 48-h exposure, the cells consumed more glucose when exposed to 100 nM OTA. Numerically, the glucose used seems to be almost completely converted into lactate. This implies that the cellular energy demand is covered not only by mitochondrial ATP production but to a substantial portion by glycolysis, leading to enhanced glucose consumption in order to guarentee the cellular energy demand. This is underlined by the observation that after 48 h the glucose in the media is almost completely used up. Additionally, the intracellular glycogen content was reduced after 48-h OTA exposure (Fig. 1c). underlining the enhanced glucose demand when OTA is present.

### Mitochondrial function

**Fig. 2:**
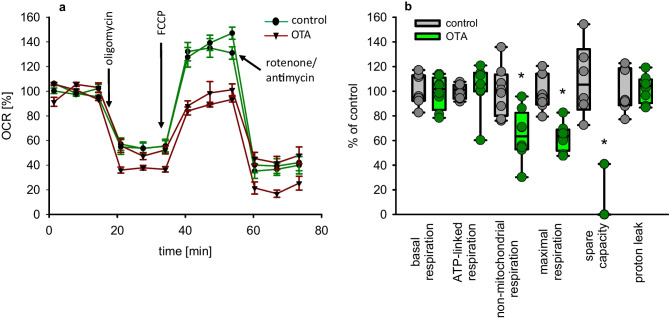
**a** Normalized oxygen consumption rate of HK2 cells after 24-h exposure to 100 nM OTA. Shown are two curve progressions of two passages obtained during the various maneuvres. **b** Results of three independent experiments showing the basal, the ATP-linked, the non-mitochondrial, the maximal mitochondrial respiration, the spare capacity and the proton leak. *N* = 3, *n* = 7–8. *p < 0.05 to the resp. controls

The increased amount of lactate after OTA exposure might be due to enhanced glycolysis and/or to reduced mitochondrial function. To get an impression in how far mitochondria are functionally active, we determined the oxygen consumption rate (OCR) of the cells using the Agilent Seahorse system. As shown in Fig. 2, the maximal respiration dropped to 63% when cells had been exposed for 24 h to OTA. Also, the non-mitochondrial respiration was lowered to 65% and the spare capacity was almost zero. The other parameters “basal respiration” and “ATP-linked respiration,” however, were not altered. Also, the proton leak was not altered. This shows that the mitochondria are not damaged unspecifically and that under basal conditions, mitochondria can supply ATP but that OTA effectively prevents an increased mitochondrial activity, which might be required in stress situations. In the case of no adaptable mitochondria, the cells must try to cope an impending energy lack by recruiting alternative ATP-producing mechanisms as anaerobic glycolysis. This insufficient reactiveness might explain the above depicted enhanced glucose demand and lactate production due to OTA exposure.

### Relation between mitochondrial and nuclear DNA copy number

**Fig. 3:**
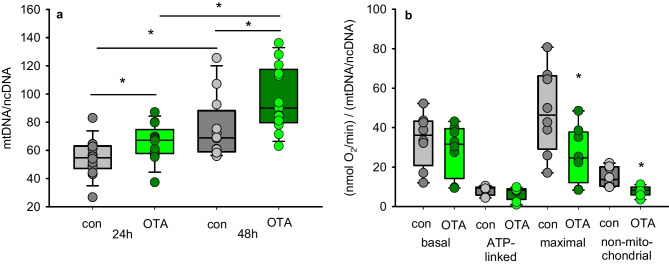
**a** Ratio of mitochondrial to nuclear DNA copy number in HK2 cells after exposure to 100 nM OTA. **b** Oxygen consumption rates related to the mt/ncDNA-ratio (24 h). *N* = 5, *n* = 12–14. *p < 0.05

To get an impression in how far the mitochondrial content of the cells may be influenced by OTA exposure, we determined the mitochondrial and nuclear DNA copy number. The ratio between mitochondrial and nuclear DNA copy number gives a valuable information about the amount of mitochondrial DNA per cell (Popov LD 2020). As shown in Fig. 3a, exposure to OTA led to an enhanced mtDNA/ncDNA ratio already after 24 h which is even more distinct after 48 h. This may indicate that the cells might respond to OTA-induced mitochondrial impairment by trying to increase the number of mitochondria. When the ratio mtDNA to ncDNA was related to the abovementioned oxygen consumption rates, it turns out that although the mtDNA was enhanced, the maximal and the non-mitochondrial OCR were still reduced after 24 h (Fig. 3b).

### Mitochondrial transcription

**Fig. 4:**
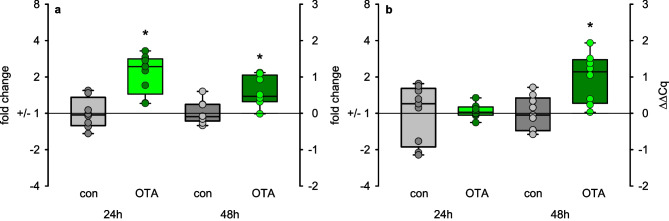
Expression of mRNA coding for TFAM (**a**) or POLRMT (**b**) in HK2 cells after exposure to 100 nM OTA. *N* = 3, *n* = 8–9. *p < 0.05 compared to resp. control

As the mitochondrial function seems to be influenced by OTA and earlier observations pointed to impaired expression of mRNAs coding for mitochondrial proteins as mitochondrial ribosomal and complex 1 proteins (Schwerdt et al. 2021), we had a look in how far also the mitochondrial transcription machinery might be influenced by OTA. TFAM is the only mitochondrial transcription factor. Interestingly, the expression of the mRNA coding for TFAM was enhanced after OTA exposure already after 24 h (Fig. 4a). The expression of the mRNA coding for the mitochondrial polymerase POLRMT was enhanced after 48-h OTA exposure (Fig. 4b). This, together with the above-described increased mitochondrial DNA copy number, may indicate that the mitochondria seem to try to counteract the OTA effect by an increased abundance.

### Expression of energy- and glucose-related proteins

**Fig. 5:**
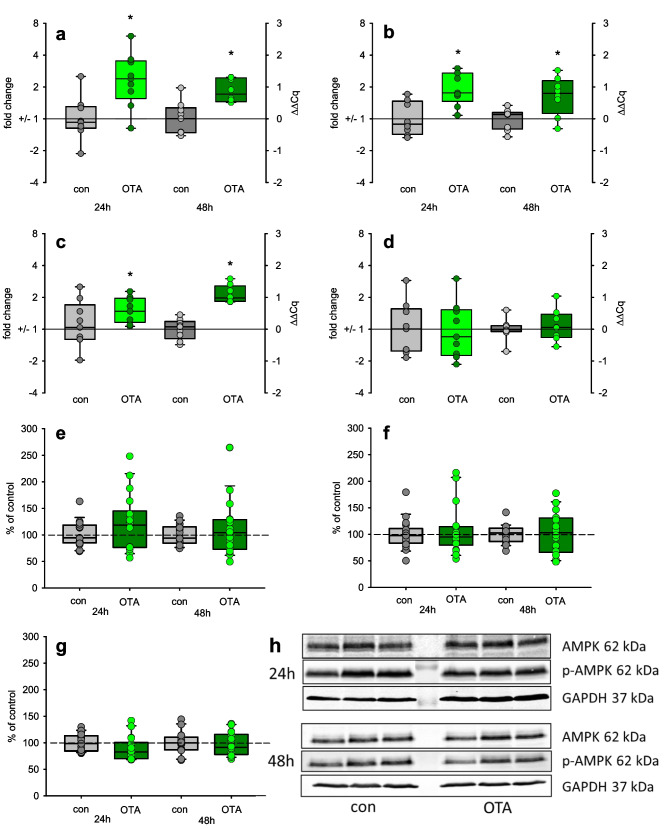
**a**–**d** mRNA expression of AMPK subunits alpha 1 (**a**), alpha 2 (**b**), beta 1 (**c**), and beta 2 (**d**) after exposure to 100 nM OTA. *N* = 3,* n* = 8–9. Protein expression of unphosphorylated (**e**) and phosphorylated (**f**) AMPK and their ratio (**g**) after exposure to 100 nM OTA. **h** Representative western blots. *p < 0.05 compared to resp. control

AMP-activated protein kinase (AMPK) serves as energy sensor and is activated at low cellular energy levels. Therefore, we determined the expression of AMPK on mRNA and protein level. As shown in Fig. 5, the mRNA expression of the three subunits (alpha 1 and 2, beta 1) was clearly increased after 24- and 48-h OTA exposure. Interestingly, this increased mRNA expression is not mirrored on protein level (Fig. 5e–h). Neither the amount of unphosphorylated AMPK was enhanced nor the amount of phosphorylated. Also, the ratio phosphorylated to unphosphorylated AMPK as an indicator of its activation was not altered. This might indicate that the cellular energy homeostasis is still balanced by the enhanced glycolytic glucose utilization and more mitochondria/cell.

**Fig. 6:**
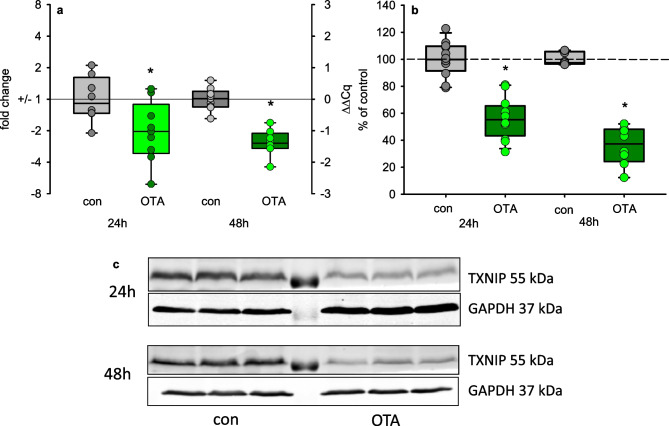
mRNA (**a**) and protein (**b**) expression of TXNIP after exposure to 100 nM OTA. **c** Representative western blots. *N* = 3–4, *n* = 8–12. *p < 0.05 compared to resp. control

One target of AMPK is TXNIP, which becomes degraded upon AMPK activation. Although we did not find an activation of AMPK, we studied the mRNA and protein expression of TXNIP. As shown in Fig. 6, not only the amount of mRNA coding for TXNIP was decreased after OTA exposure but also the TXNIP protein amount was lowered.

**Fig. 7:**
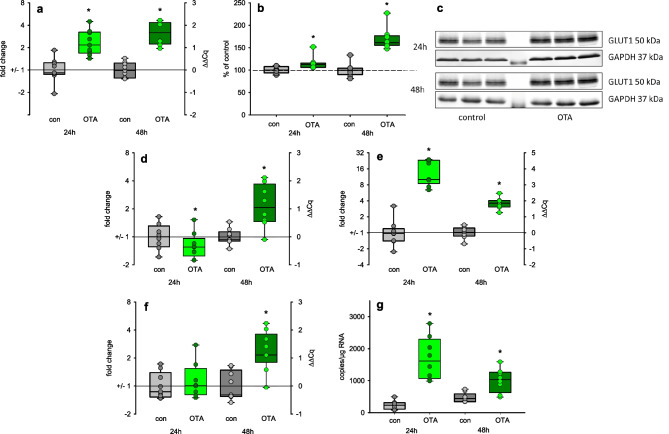
Expression of glucose-related proteins: **a** expression of mRNA coding for GLUT 1 (SLC2A1), **b** protein expression of GLUT1, **c** representative western blots of GLUT1; **d** expression of mRNA coding for GLUT3 (SLC2A3), for SGLT2 (SLC5A2; **e**), for hexokinase (HK2, **f**), and for uncoupling protein (UCP1, **g**) after exposure to 100 nM OTA. *N* = 3, *n* = 8–9. *p < 0.05 compared to resp. control

One target of TXNIP is the passive glucose transporter GLUT1 (SLC2A1). We already have shown that the expression of the mRNA coding for GLUT1 was enhanced after OTA exposure (Schwerdt et al. 2021). Here, we additionally studied the GLUT1 protein expression. As shown in Fig. 7, besides the increased mRNA expression, also the GLUT1 protein level was increased after OTA exposure and staining the cells for GLUT1 also showed an increased abundance of GLUT1 after OTA exposure (supplements). Also, the expression of mRNA coding for GLUT3 was enhanced, although only after 48 h (Fig. 7d) whereas the mRNA expression of the secondary-active glucose transporter SGLT2 was clearly enhanced already after 24 h (Fig. 7e). The glucose imported is phosphorylated by hexokinase. The expression of mRNA coding for hexokinase is also enhanced after 48 h (Fig. 7f). Also, the expression of the mRNA coding for the uncoupling protein (UCP1) is enhanced contributing to insufficient mitochondrial ATP production (Fig. 7g) The up-regulation of the glucose transporters enables the cell to import more glucose and to cover an eventual enhanced glucose demand induced by impaired mitochondrial function due to OTA exposure.

## Discussion

Ochratoxin A (OTA) is a mycotoxin found in a variety of human foodstuff (Malir et al. 2016; Marin et al. 2013), and therefore, its daily uptake is almost unavoidable. OTA has nephrotoxic potential in animals and is probably involved in human kidney diseases (Fuchs and Peraica 2005; Pfohl-Leszkowicz and Manderville 2007). Since its description in 1965 by van der Merve et al. (van der Merwe et al. 1965), many studies have been performed to unveil the mode of action of OTA (Heussner and Bingle 2015) and a bright bundle of possible mechanisms have been suggested as e.g. disturbance of protein synthesis (Dirheimer and Creppy 1991), apoptosis (Schwerdt et al. 1999), interaction with signaling pathways, formation of DNA adducts (Mally and Dekant 2005) and/or reactive oxygen species (Tao et al. 2018), as well as interaction with post-transcriptional processes (Chen et al. 2022; Zhu et al. 2017). Despite all these findings, the “real” responsible mechanism is still not known and—maybe—it is not only one but the interplay of more than one of these factors.

In the present study, we want to add another possible player into the discussion: mitochondria, respectively the resilience of cellular energy homeostasis. An earlier study has already described that mitochondria might play a role in the effects of OTA, showing that an OTA-induced acidification depends on mitochondria (Eder et al. 2000). Starting from the observation that after longer OTA-exposure renal proximal tubule cells show a quantitatively equivalent lactate production, we asked ourselves what might be the reason for this enhanced glucose consumption? Glucose can be used in the cell to produce ATP by two mechanisms: a rather ineffective glycolysis with production of lactate or oxidative phosphorylation, involving mitochondria. This presumes intact mitochondria.

Besides supplying the cell with ATP, mitochondria additionally play a role in ROS formation (ROS as one of the supposed mode of OTA-action) and apoptosis, another effect of OTA (Gekle et al. 2000). Intact mitochondria are dependent on a correct protein supply from the cell as most of mitochondrial proteins are coded in the cell nucleus but they also require their own protein synthesis machinery including ribosomal subunits. In a previous study using human proximal tubule cells (HK2), we have already observed a lowered mRNA expression not only of mitochondrial ribosomal subunits but also of mitochondrial proteins of the respiratory chain after OTA exposure (Schwerdt et al. 2021). In that study, we also observed that the mRNA expression of glucose-handling enzymes was altered in a way that indicates a higher utilization of intracellular glucose stores. In the present study, we could confirm this idea by showing that the intracellular glycogen content was almost used up after 48-h OTA exposure (as was the glucose in the media). This underlines the assumption that the cells are forced to use enhanced glucose amounts, probably because the mitochondria cannot supply enough energy.

We studied the mitochondrial function by determining the oxygen consumption rate after OTA exposure. The unaltered proton leak excludes an unspecific, mitochondria-damaging effect of OTA. Also, the basal and the ATP-linked respiration was not altered by OTA indicating that under basal conditions the mitochondria work properly also in the presence of OTA. But the maximal respiration was clearly diminished as well as the spare capacity. This indicates that a basic turnover might be possible but that there is no additional capacity to react to a higher energy demand, which might be the case in cellular stress situations. Also, the non-mitochondrial oxygen consumption was reduced indicating that OTA influences other oxygen-consuming reactions as e.g. those of oxidases. Interestingly, the mitochondrial DNA copy number increases when cells were exposed to OTA but the maximal and the non-mitochondrial oxygen consumption rate related to mtDNA/nclDNA were still reduced. This might reflect a kind of reaction of the mitochondria in order to re-establish mitochondrial function which, however, is not sufficient to secure an enhanced energy demand. The enhanced mRNA expression of the mitochondrial transcription factor TFAM and of the mitochondrial polymerase POLRMT may support this idea.

The cellular energy homeostasis is a well-balanced challenge. Mechanisms involved therein include the AMP-activated kinase. AMPK is activated by lowered energy situations mirrored in increasing AMP/ATP ratios. AMPK helps the cells to survive low energy conditions by turning on energy-generating pathways and lowering energy-consuming processes. Additionally, AMPK has an important role in mitochondrial homeostasis (Glosse and Föller 2018; Juszczak et al. 2020). We found that not only the amount of unphosphorylated and phosphorylated AMPK was not altered by OTA exposure although the mRNA was clearly enhanced but also the extent of phosphorylation was unchanged. The discrepancy between mRNA and protein level might point to posttranslational mechanisms involved in regulation of AMPK regulation (Bronisz et al. 2015). On a first view, this might indicate that the cellular energy level is not endangered by OTA and therefore not claiming for AMPK-involving reactions. It might also be that for a long time the cells are able to prevent an increased AMP/ATP ratio by enhanced usage of anaerobic pathways. Or that the AMPK has already done its job to lower the overall energy consumption and to increase the glucose supply.

Another player in glucose regulation is the thioredoxin-interacting protein, TXNIP. TXNIP is degraded via AMPK upon energy stress and is an important regulator for glucose homeostasis as it suppresses glucose uptake by internalization of glucose transporter and reducing their mRNA expression (Wu et al. 2013; Yoshihara 2020). We found a lowered TXNIP expression on the mRNA as well as on the protein expression level although the AMPK activity was unaltered. The lowered expression of TXNIP can explain the enhanced expression of GLUT1 both on mRNA and on protein level observed after OTA exposure. Also, the increased expression of mRNAs coding for the glucose transporters GLUT3 and SGLT2 and of the first enzyme in cellular glucose utilization, hexokinase, indicates that the cells try to establish a higher glucose import. To which amount the different glucose transporter may contribute to glucose uptake remains unknown. The enhanced glucose transport is needed to fuel the ineffective anaerobic ATP generation as the mitochondria seem to fail to guarantee a sufficient energy supply, which might be supported by enhanced uncoupling.

In summary, here we present hints, which might make it worthwhile to have a closer look on the role of energy homeostasis and of mitochondria in the OTA-induced scenario in cells. In a recent study using a hepatoma-derived cell line (HepG2), an influence of OTA on the metabolome has been shown (Gerdemann et al. 2022). In how far these findings can be transferred to kidney tubule cells and in how far other cells as fibroblasts interacting with tubule cells (Schwerdt et al. 2021) may influence the described scenario has to be investigated in further studies.

### Supplementary Information

Below is the link to the electronic supplementary material.

Supplementary file1 (DOCX 1829 KB)

## Data Availability

Data are available from the author on request.

## Abbreviations

OTA: Ochratoxin A
AMPK: AMP-activated protein kinase
TXNIP: Thioredoxin-interacting protein
OCR: Oxygen consumption rate
