# Supplementary material for: The nephrotoxin ochratoxin a impairs resilience of energy homeostasis of human proximal tubule cells
Source: Mycotoxin Res. 2023 Jul 19;39(4):393–403. doi: 10.1007/s12550-023-00500-7 (PMC10635976; doi:10.1007/s12550-023-00500-7)

Supplementary material

Staining of GLUT1 in HK2 cells

HK2 cells were seeded onto poly-L-lysine-coated coverslips and exposed for 24 hours to 100 nM OTA. Cells were fixed by 15 min incubation with 4% paraformaldehyde. After washing with PBS cells were permeabilized by 1% TritonX-100 (30 min), washed again (1 x 10 min with PBS/1% SDS/100 mM glycine, 1 x 10 min with PBS/100 mM glycine) and blocked by PBS/10% goat serum/1% BSA. Primary antibody (diluted 1:300 in PBS/1% goat serum/1% BSA; Abcam ab10214) was added overnight. After washing with PBS, 2nd antibody was added (Oregon Green-coupled anti-rabbit, 1:1000 in PBS/1% goat serum/1% BSA; Invitrogen O-11038) for one hour. To stain the nuclei, after washing with PBS, DAPI solution (4´,6-diamino-2-phenylindole; 1 µg/ml) was added for 2 minutes and again washed with PBS.

Supplement figures: representative pictures of two coverslips. Green: GLUT1. Blue shows cell nuclei (DAPI-stained). 40 x magnification, white bar is 100 µm. The middle graphics show surface blots of the green stained GLUT1


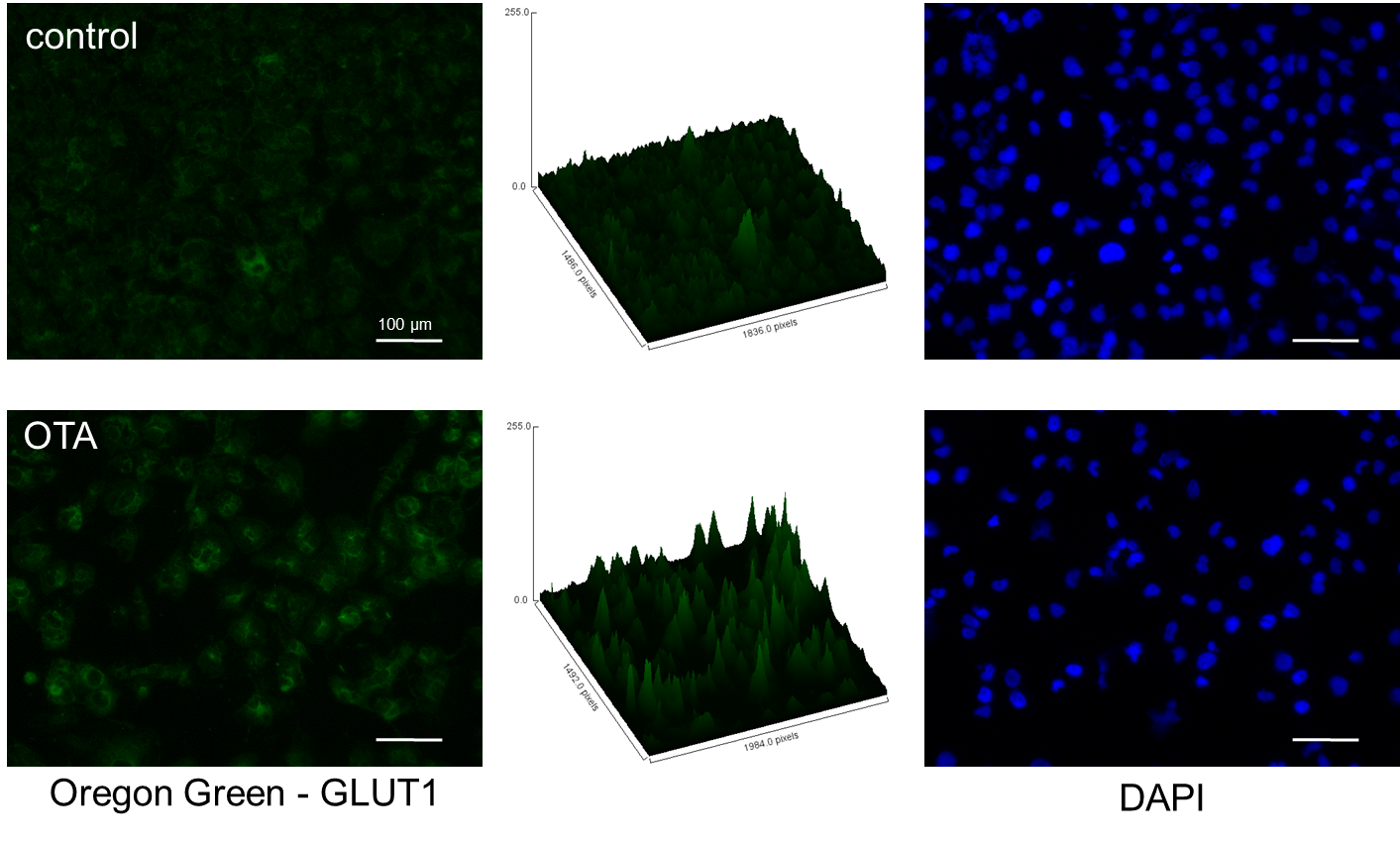


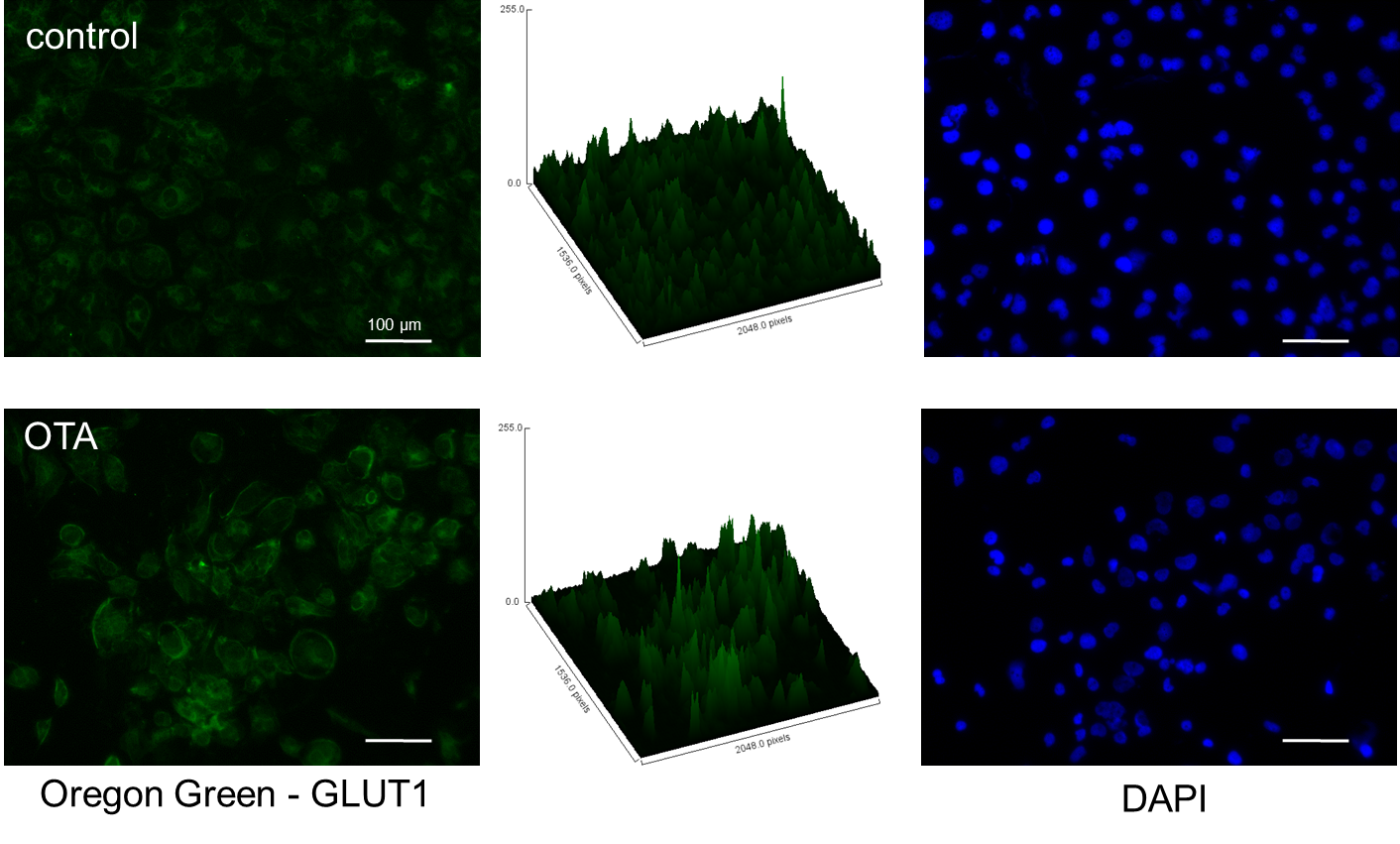

Supplement: Supplementary file 1 — Supplementary file1 (DOCX 1829 KB) [file 12550_2023_500_MOESM1_ESM.docx]
